# Insight into Bone-Derived Biological Apatite: Ultrastructure and Effect of Thermal Treatment

**DOI:** 10.1155/2015/601025

**Published:** 2015-01-28

**Authors:** Quan Liu, Haobo Pan, Zhuofan Chen, Jukka Pekka Matinlinna

**Affiliations:** ^1^Dental Materials Science, Faculty of Dentistry, The University of Hong Kong, Pokfulam, Hong Kong; ^2^Shenzhen Key Laboratory of Marine Biomaterials, Shenzhen Institute of Advanced Technology, Chinese Academy of Science, Shenzhen 518055, China; ^3^Department of Oral Implantology, Hospital of Stomatology, Guanghua School of Stomatology, Institute of Stomatological Research, Guangdong Provincial Key Laboratory of Stomatology, Sun Yat-sen University, Guangzhou 510055, China

## Abstract

*Objectives*. This study aims at examining the ultrastructure of bone-derived biological apatite (BAp) from a series of small vertebrates and the effect of thermal treatment on its physiochemical properties. *Materials and Methods*. Femurs/fin rays and vertebral bodies of 5 kinds of small vertebrates were firstly analyzed with X-ray microtomography. Subsequently, BAp was obtained with thermal treatment and low power plasma ashing, respectively. The properties of BAp, including morphology, functional groups, and crystal characteristics were then analyzed. *Results*. The bones of grouper and hairtail were mainly composed of condensed bone. Spongy bone showed different distribution in the bones from frog, rat, and pigeon. No significant difference was found in bone mineral density of condensed bone and trabecular thickness of spongy bone. Only platelet-like crystals were observed for BAp obtained by plasma ashing, while rod-like and irregular crystals were both harvested from the bones treated by sintering. A much higher degree of crystallinity and larger crystal size but a lower content of carbonate were detected in the latter. *Conclusion*. Platelet-like BAp is the common inorganic component of vertebrate bones. BAp distributing in condensed and spongy bone may exhibit differing thermal reactivity. Thermal treatment may alter BAp's *in vivo* structure and composition.

## 1. Introduction

Nowadays, certain animal bone is one of the main sources of commercial xenogeneic grafting materials used for bone reconstruction in implant dentistry, maxillofacial surgery, and orthopedics [1–3]. Because vertebrate bones have a common inorganic component, that is, biological apatite (BAp), the clinical effect of such commercial products is highly related to its micro- and nanostructure as well as chemical composition. Nowadays, animal bones, such as bovine and porcine bones that demonstrate high similarity in microstructure and remodeling to that of human bones have been used for the preparation of grafting products [4, 5]. Despite the outstanding clinical outcome of such commercial grafting materials, their drawbacks such as a relatively low rate of biodegradation cannot be ignored and they remain a challenge to be resolved, because such delayed bioresorption of grafting materials may have a negative effect on the process of bone reconstruction [6]. As in a consistent biological environment, bioresorption rate depends on the physiochemical properties of the grafting material itself, such as morphology, crystal size, crystallinity, and chemical composition. Such slow bioresorption may indicate that either the understanding of the properties of bone-derived BAp is incomprehensive or the treatment process of BAp preparation has a negative influence on its properties, or both.

Regarding to BAp's morphology, as an example, platelet-like, rod-like, and irregular crystals of BAp were reported in literature [4, 7–9]. To some extent, this inconsistent finding may be an artifact resulting from various treatment approaches that probably have an unknown influence on BAp's properties [4]. As the most common way to obtain BAp by eliminating the bone matrix, a thermal treatment where a high temperature is involved may cause alterations to the physiochemical properties of BAp. In fact, the influence of thermal treatment on the physiochemical properties of human and bovine bone-derived BAp has been investigated and reported elsewhere [10, 11]. The crystal size and crystallinity were found to increase with sintering temperature. However, some drawbacks in those studies cannot be denied, such as limitations in the research object source and sample size, lack of comparison between the properties of pre- and posttreated BAp from the same source. With low power plasma ashing that is believed to have no significant effect on the* in vivo* properties of BAp, Kim et al. [8] reported that BAp derived from a series of animal bones has a very thin platelet contour with a wrinkled edge, which provides a blank control for the former studies. However, it is inappropriate to simply combine and interpret their findings since they had different bone sources. Given this, both the* in vivo* nanostructure of BAp and the effect of thermal treatment on its physiochemical properties have not yet been fully understood.

This said, in the current study, the physiochemical properties, especially the micro- and nanostructure of BAp derived from a series of small vertebrates' bones were investigated. Two different treatment approaches were applied, that is, low power plasma ashing and thermal treatment, aiming at achieving some insight into the* in vivo* structure of BAp as well as the possible effect of thermal treatment.

## 2. Materials and Methods

A total of 20 (5 of each kind) fresh carcasses of fish (hairtail (*Trichiurus lepturus*): ~650 g; grouper (*Epinephelus tauvina*): ~300 g), frogs (*Rana temporaria*, ~100 g) and pigeons (*Streptopelia risoria*, ~500 g) were obtained from a local market, while 5 fresh carcasses of Sprague-Dawley rats (*Rattus norvegicus*, ~3 months, ~330 g) were donated by the Laboratory Animal Unit (where the rats were bred) of Southern Medical University (Guangzhou, China). Femurs/fin rays and vertebral bodies (thoracic vertebra) were harvested. After cleaning the macroscopic impurities such as soft tissues and blood, bone samples were stored by immersing in absolute ethanol (99.7%, AnalaR grade, BDH, Poole, UK).

### 2.1. Micro-CT Scanning of Bones

In total, 50 bones (5 kinds of animals × 5 individuals × 2 bone types mentioned above) were examined with microcomputed tomography (*μ*-CT, Skyscan1076* in vivo* X-Ray Microtomograph, Skyscan, Kontich, Belgium) [12] for the microstructure observation and analysis. The parameters for *μ*-CT scanning were as follows: 59 kV, 0.5 mm Al filter, 149 *μ*A source current, 8.665 *μ*m isotropic resolution, 2400 ms exposure time, and 0.800° rotation step. The obtained raw images were reconstructed with NRECON software (v.1.6.6, Skyscan, Kontich, Belgium) according to the following parameters: standard reconstruction mode, connected reconstruction (parts) = 3, section to section step = 1. Based upon such images, a different site of bones was identified and classified into condensed and spongy bone, accordingly. Afterwards, the properties of the two groups of bone were further analyzed by using CTAn software (v.1.1.1, Skyscan, Kontich, Belgium), respectively [13]. In detail, the bone mineral density (BMD, g/cm^3^) was firstly measured for both condensed and spongy bone after calibrating the software with two hydroxyapatite phantoms (density: 0.25 and 0.75 g/cm^3^, resp.). As to condensed bone, 100 slices in the middle part of each bone site were chosen as the region of interest (ROI, thresholding: 90), and the indices including the total cross-sectional area (Tt.Ar, mm^2^), condensed bone area (Ct.Ar, mm^2^), condensed thickness (Ct.Th, mm), and condensed bone fraction (Ct.Ar/Tt.Ar, %) were measured in a two-dimensional mode (2D-mode). For spongy bone, 100 slices from the 201st to 300th slice starting from the distal end of the bone site were adopted as the ROI (thresholding: 90). Upon this ROI, a further region including spongy bones only was determined manually along the inner surface of condensed shafts. Afterwards, a three-dimensional (3D) analysis for the indices including bone volume density (bone volume/tissue volume, BV/TV, %), bone surface density (bone surface/tissue volume, BS/TV, mm^−1^), mean trabecular thickness (Tb.Th, mm), mean trabecular number (Tb.N, mm^−1^), and mean trabecular separation (Tb.Sp, mm) was done for the spongy bone [14].

### 2.2. Preparation of BAp

#### 2.2.1. Low Power Plasma Ashing

As the control, a total of 20 bones (5 kinds of animals × 2 individuals × 2 bone types) were cut into pieces and further treated with low power plasma ashing (K1050 plasma asher, Quorum Technologies, Lewes, East Sussex, UK) [8]. Briefly, the bone pieces were placed in clean ceramic crucibles and fixed on a sample holder, which was at the center of the reaction chamber filled with ionized oxygen. A pretreatment of ashing (75 w, 2 h) was carried out to remove the adsorbed ethanol, residual water, and part of the organics. The obtained bone pieces became brittle and were next ground into powder manually so as to enlarge the contact surface between bone and oxygen. Ultrasonication (100 w, 5 min) and low-speed centrifugation (1000 rpm, 5 min) in ethanol were used to separate the crystals from the bone residue. The bone residue was dried in air and then further plasma-ashed (75 w, 4 h) for 5 periods with intervals for ultrasonication and low-speed centrifugation. All the supernatant was collected and centrifuged at high speed (10000 rpm, 5 min) to obtain a pellet of crystals, which was then plasma-ashed (75 w, 6 h) to remove the organic residue. During the whole process of low power plasma ashing, the peak temperature in the reaction chamber was around 140°C.

#### 2.2.2. Thermal Treatment

Another lot of 30 bones (5 kinds of animals × 3 individuals × 2 bone types) was sintered to eliminate the organic matrix. In detail, the bones were placed in clean ceramic crucibles and calcinated at 800°C for 2 h (heating rate: 10 K min^−1^) in an electric furnace (Vulcan 3–550, Dentsply International, Pennsylvania, USA). When the sample cooled down (cooling rate: 10 K min^−1^) to room temperature, the obtained bone ash was ground into powder manually and kept in a desiccator over silica gel for further tests.

### 2.3. Characterization of BAp

For the samples obtained by the two methods described above, the crystal morphology and size were examined with a scanning electron microscope (SEM) (S-4800, Hitachi Science Systems, Tokyo, Japan) and a transmission electron microscope (TEM) (Tecnai G2 20, FEI Corporation, GG Eindhoven, The Netherlands). For the SEM analysis, the bone powder was cemented on copper stubs by using a conductive graphite tape and next they were sputter-coated with gold for 40 s before observation at 5 kV. As to the TEM test, the sample was further ground in absolute ethanol and a pipette drop of the mixture was dispersed onto a Formvear-coated, carbon-reinforced copper grid for direct examination without further treatment. The crystal characteristics were examined with X-ray diffraction (XRD) (D/max III 2200 V/PC, Rigaku, Tokyo, Japan). The powdered samples were mounted on glass stubs and tested from 4° to 60° (2*θ*) angles at a scanning step of 0.02° and scanning speed 4° min^−1^. The XRD patterns were analyzed with MDI Jade software (v.5.0, Materials Data, CA, USA), incorporated with PDF cards (PDF2-2004, International Centre for Diffraction Data (ICDD), Newton Square, PA, USA). The functional groups of the bone samples were identified by using Fourier-transform infrared spectroscopy (FTIR) (Vector 33, Bruker Optics, Ettlingen, Germany). Powdered samples were firstly mixed (1 : 100) with KBr (IR grade, Merck, Giessen, Germany) and manually ground by using an agate mortar and pestle and then uniaxially compressed (10 MPa) into pellets for examination. The IR spectra were collected using the transmittance mode with a scanning range of 4000–400 cm^−1^.

### 2.4. Data Analysis

All quantitative data were analyzed with one-way ANOVA (SPSS, v.16.0, IBM, NY, USA) at a significance level of 0.05 (*α* = 0.05).

## 3. Results

### 3.1. Micro-CT

**Figure 1:**
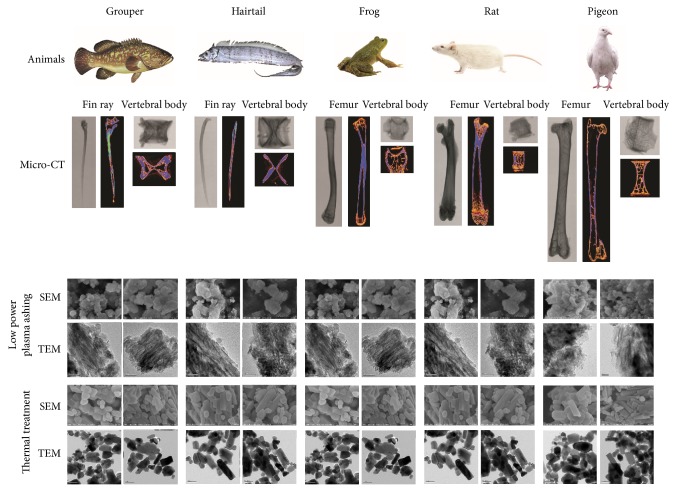
Ultrastructure of animal bones with two preparation approaches.

As shown in Figure 1, bone trabeculae were observed in the bones of frog, rat, and pigeon instead of grouper and hairtail. According to the color map automatically drawn to label condensed (blue) and spongy bone (orange) roughly, the fin rays and vertebral bodies of grouper and hairtail were mainly composed of condensed bone, where rare spongy bone was detected, although there were some pores in the bones. So was the middle part of femurs that were marked in blue. By contrast, spongy bone presented in orange was distributed in the both ends of femurs and vertebral bodies of frog, rat, and pigeon.

#### 3.1.1. BMD

**Figure 2:**
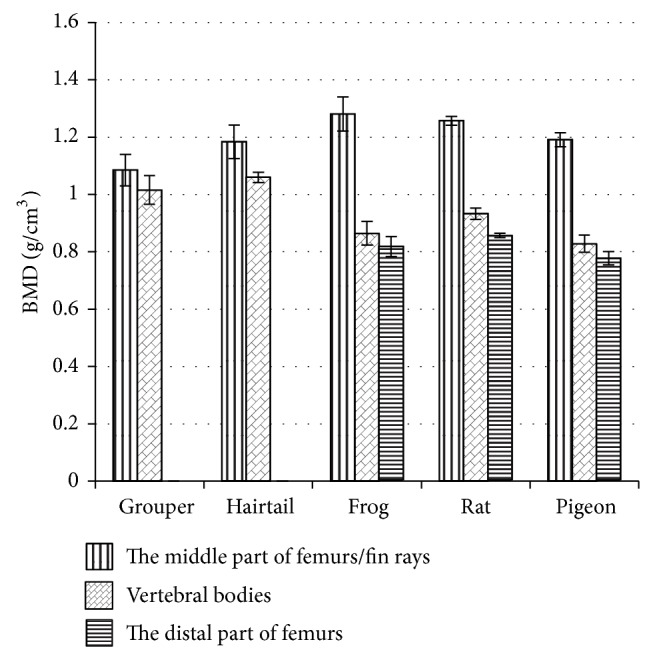
Bone mineral density (BMD) of animal bones.

At the significance level of 0.05, for the comparison of the middle part of femurs/fin rays, grouper showed the lowest BMD (Figure 2; 1.085 ± 0.055 g/cm^3^, *P* = 0.002), but no significant difference was found in the rest. As to the vertebral body, the two fish had a similar BMD (Figure 2; grouper: 1.016 ± 0.050 g/cm^3^; hairtail: 1.060 ± 0.018 g/cm^3^), which was close to the middle part of femurs/fin rays and significantly higher than the rest (*P* < 0.001), while pigeon had the lowest BMD (0.828 ± 0.030 g/cm^3^;  *P* < 0.001). In the distal part of femurs, the BMD of frog (0.813 ± 0.035 g/cm^3^) and rat (0.851 ± 0.008 g/cm^3^) were close and significantly higher than that of pigeon (Figure 2; 0.772 ± 0.023 g/cm^3^; *P* = 0.001).

#### 3.1.2. Spongy Bone

**Figure 3:**
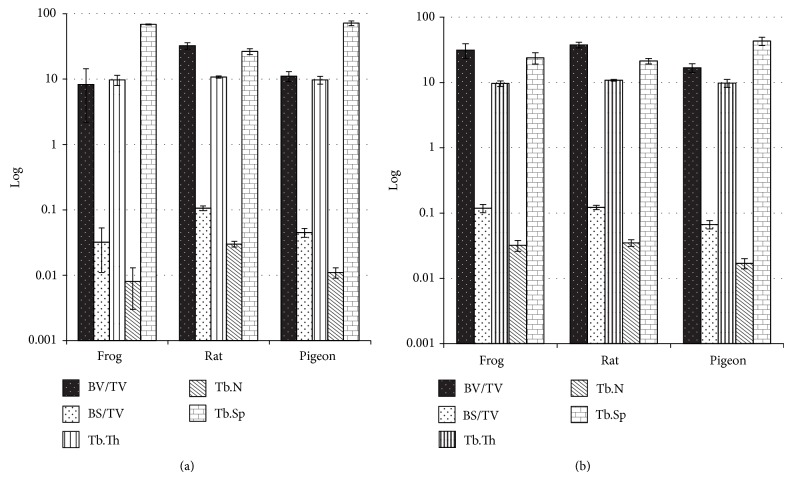
Comparison of spongy bones among animals: BV/TV, BS/TV, Tb.Th, Tb.N, Tb.Sp ((a) the distal part of femurs; (b) vertebral bodies).

There was no statistical difference in Tb.Th of either the distal part of femur (*P* = 0.346, *α* = 0.05) or vertebral body (*P* = 0.108, *α* = 0.05) among frog, rat, and pigeon. In the distal part of femur, rat had significantly higher BV/TV (32.311 ± 3.804%; *P* = 0.008), BS/TV (0.106 ± 0.009 mm^−1^; *P* < 0.001), and Tb.N (0.030 ± 0.003 mm^−1^; *P* = 0.008) but lower Tb.Sp (26.445 ± 2.702 mm; *P* = 0.009) than frog and pigeon (Figure 3(a)). In vertebral body, pigeon showed the lowest BV/TV (16.782 ± 2.556%; *P* = 0.006), BS/TV (0.067 ± 0.010 mm^−1^; *P* < 0.001), and Tb.N (0.017 ± 0.003 mm^−1^; *P* < 0.001) but the highest Tb.Sp (43.105 ± 6.167 mm; *P* < 0.001) among the three animals (Figure 3(b)).

#### 3.1.3. Condensed Bone

**Figure 4:**
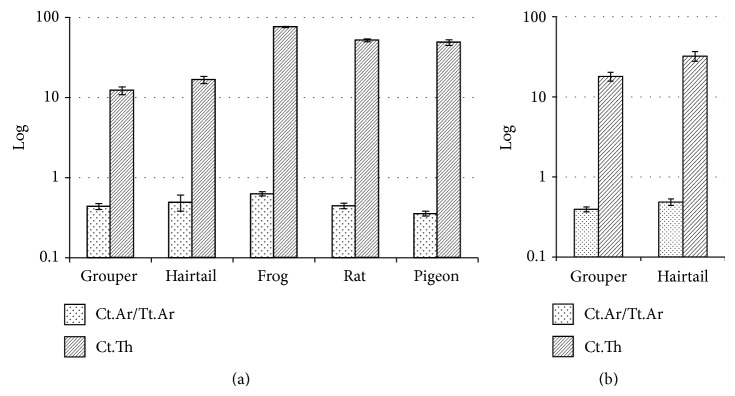
Comparison of condensed bones among animals: Ct.Ar/Tt.Ar, Ct.Th ((a) the middle part of femurs/fin rays; (b) vertebral bodies).

In the middle part of femurs/fin rays, frog had statistically higher Ct.Ar/Tt.Ar and Ct.Th than grouper, hairtail, rat, and pigeon (Tables 1 and 2; Figure 4(a); *P*_Ct.Ar/Tt.Ar_ = 0.003, *P*_Ct.Th_ < 0.001). No significant difference was found among the latter four. Besides, hairtail showed significantly higher value than grouper in both Ct.Ar/Tt.Ar (*P* = 0.005) and Ct.Th (*P* < 0.001) of vertebral body (Figure 4(b)).

### 3.2. Nanostructure

#### 3.2.1. SEM/TEM

In all the specimens treated by low power plasma ashing, no rod-like crystals but irregular platelets were observed to be piled together, which were more distinguishable in the TEM fields. Artifacts of rod-like shape resulted from such pileups and overlaps could be seen in some parts of the images. However, the size of crystals can hardly be determined both in the SEM and in the TEM images, although most of the crystals seemed no larger than 100 nm (Figure 1).

In the thermal-treated samples, crystal aggregation was detected. Despite this, only irregular platelet-like crystals were observed in the fin rays and vertebral bodies of grouper and hairtail (Figure 1). The crystal size was around 100~200 nm in grouper and 100~400 nm in hairtail. Both such irregular crystals and short rod-like ones could be observed in frog femur and vertebral body, in a size of 100~200 nm (width) and 200~400 nm (length). Long rod-like crystals around 100~200 nm wide and 700~900 nm long were found in frog femur. Such long rod-like crystals were the dominant form for rat and pigeon, especially in the femurs, although their size varied in a larger range compared with those in frog.

#### 3.2.2. XRD

**Figure 5:**
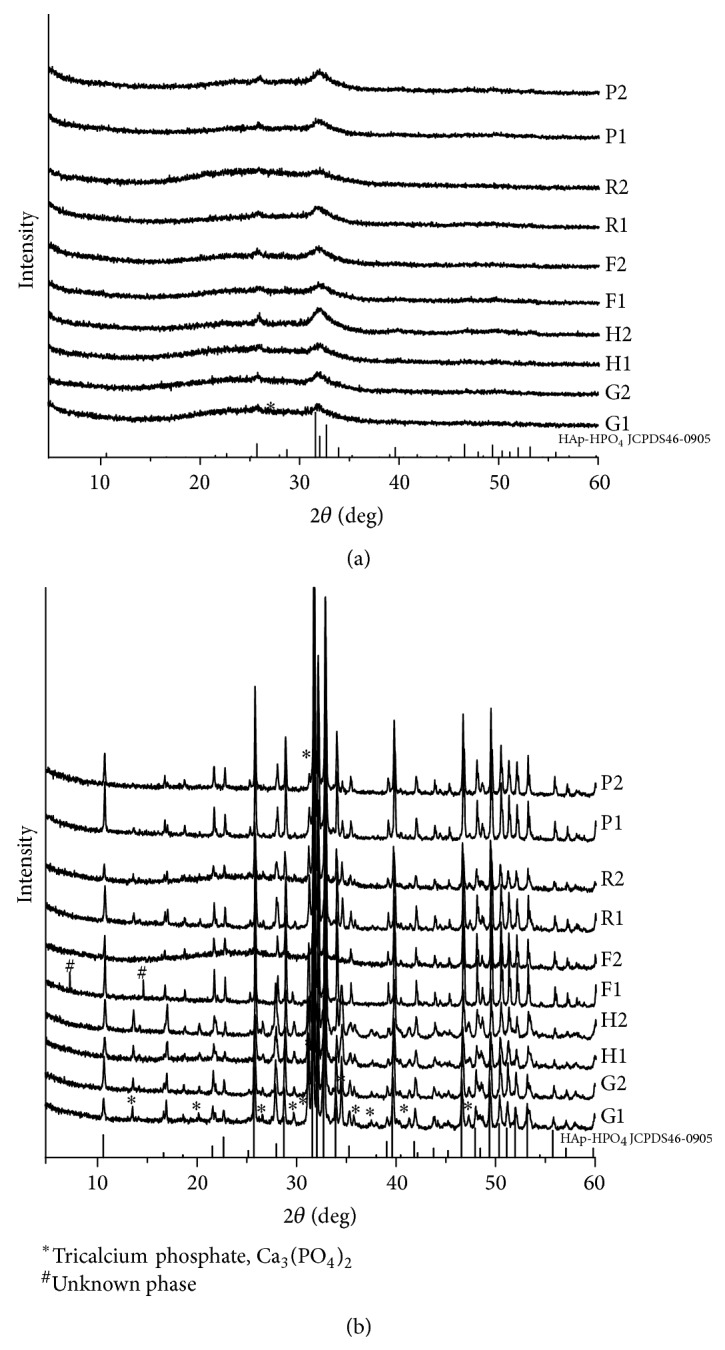
XRD patterns of BAp ((a): low power plasma ashing; and (b) thermal treatment; 1 = femur/fin ray, 2 = vertebral body; G = grouper, H = hairtail, F = frog, R = rat, P = pigeon).

After sintering, the major XRD patterns of grouper, hairtail, rat, and pigeon were fitted with that of Ca_9_HPO_4_(PO_4_)_5_OH, while frog femur and vertebral body showed a particular XRD pattern of Ca_10_(PO_4_)_5_CO_3_(OH)F and Ca_5_(PO_4_)_3_OH, respectively. In addition, some peaks pertaining to minor phases of bivalent cations (Co^2+^, Fe^2+^, Mn^2+^, Ni^2+^) incorporated in calcium phosphate or compounds (Mn_3_N_2_) were detected as well (Table 3; Figure 5(b)). In comparison to all these well crystallized XRD patterns, all those plasma-ashed specimens were atypical, where only the representative peaks of calcium phosphate apatite at 26°and 32° (2*θ*) could be identified (Figure 5(a)).

#### 3.2.3. FTIR

**Figure 6:**
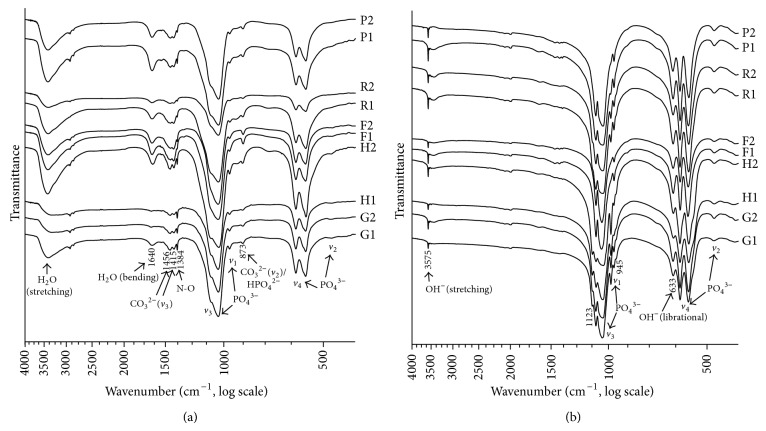
FTIR spectra of BAp ((a): low power plasma ashing; and (b) thermal treatment; 1 = femur/fin ray, 2 = vertebral body; G = grouper, H = hairtail, F = frog, R = rat, P = pigeon).

As shown in the FTIR spectra (Figure 6), absorption peaks of phosphate groups at 460, 560–610, 960 and 1020–1095 cm^−1^ were observed in all samples. However, the intensity of phosphate in the plasma-ashed samples (Figure 6(a)) was much lower than that of the thermal-treated ones (Figure 6(b)), while the peaks of adsorbed water at 2800–3600 and 1640 cm^−1^ became much weaker after sintering. The bands at 945 and 1123 cm^−1^ (Figure 6(b)) were due to the presence of HPO_4_^2−^ in the bones of grouper, hairtail, rat, and pigeon, while those at 633 and 3575 cm^−1^ were attributed to the stretching and libration of OH^−^ (Figure 6(b)). Some bands detectable in the plasma-ashed samples were absent in the thermal-treated ones, including carbonate at 873, 1415, and 1456 cm^−1^, as well as N–O at 1384 cm^−1^ (Figure 6(a)).

## 4. Discussion

### 4.1. Selection of Materials

In order to achieve a balance between a relatively large investigation scope and consistent parameters of examinations, some small representative animals from aquatics, amphibians, mammals, and birds were chosen in the present study, while large animals such as pig and cattle were not adopted although they are the main source of nowadays commercial grafting materials. In addition, these small vertebrates are easily reachable and also suitable for *μ*-CT examination, without further treatment such as cutting or dissection. *μ*-CT scanning provided a nondestructive way to capture the information of BAp's structure at microscale, which also revealed the difference in the distribution of condensed and spongy bone among the animals examined. Besides, as discussed extensively elsewhere [4], the age of BAp crystals (the time from crystal formation to extraction) is more important to their shape, structure, and chemical composition than the age of their donator, although the age of animal is related to bone quality. In each bone, BAp crystals in different age were contained, which lowered the bias from the unknown age of animals (fish, frog, and pigeon). However, this limitation is expected to be improved in further studies for vertebrates with a clear record of their age.

In addition, from a proposed evolutionary point of view, two types of bone formation are found in the evolution process of vertebrates, namely, intramembranous and endochondral ossification [15]. The former forms bones including spine, ribs, sternum, and skull, which are supposed to be older in the evolution process and may reserve the original characteristics. On the other hand, the latter is responsible for the formation of appendicular bones such as femur, tibia, and fibula, to which major changes may occur in the process of adaptation [15]. Given this, both the spines and femurs/fin rays were collected and examined in this study so as to obtain a panoramic view of the structural difference of bones among species. As shown in the *μ*-CT images, it was the femurs/fin rays that show more substantial alterations than the vertebral bodies.

### 4.2. Preparation of BAp

Many approaches have been developed and used for the extraction of BAp from bones and teeth [4], where the thermal treatment may be the most common one because of its relatively simple and controllable procedure [4, 16–18]. It was reported that the organic components could be eliminated by calcinating at 600°C [17] and there were no substantial changes in the morphology of apatite crystal from rat after being sintered at 800°C for 2 h [19]. On the contrary, the morphological and compositional changes of apatite crystals caused by a thermal treatment were observed [20–22]. However, all those conclusions were ambiguous because a blank control was lacking in those studies. By a thermal treatment which is usually carried out at more than 600°C, this goal is hardly to be achieved because BAp starts losing carbonate from crystal lattice at a temperature of 180°C or even lower [23], and it begins growing larger at around 600°C [4].

Low power plasma ashing is believed to be capable of reserving the* in vivo* characteristics of BAp [8] since it depends on the oxidation of ionized oxygen at low temperature (less than 200°C), turning organics into gases. Upon this method, Kim et al. [8] observed platelet-like crystals with wrinkled edges as the common BAp in a series of animals, including fish, chicken, cattle, and mouse. It was also used in a subsequent study to compare the physical and chemical differences of BAp from condensed and spongy bone of cattle [24]. Given this, it was utilized in this study as the control approach. With plasma ashing, platelet-like crystals were observed in all specimens, which is corresponding to some previous studies [7, 8, 25], although the reported wrinkled edges were not found. Nevertheless, low power plasma ashing is a time-consuming method. It was reported that there was still some organic residuals in the bone samples after 40 days' ashing [24]. Such residual components may be one of the causes of the crystal pileups observed in the current study.

### 4.3. Microstructure

As shown in the grey images of bones, the distribution and content of condensed and spongy bones were different among the species studied. Spongy bone was mainly found in the vertebral bodies of frog, rat, and pigeon as well as the both ends of femurs. By contrast, the vertebral bodies of fish, all the fins, and the middle part of femurs were primarily constructed by condensed bone, which was supported by further analysis, such as the color mapping as well as BMD. Depending on the image volume ratio and the setting of grey threshold, BMD reflects the density of inorganic component of bones [14]. In the current study, there seemed no dramatic diversity of BMD in either condensed boneor spongy bone, which were in the range of 1.0~1.3 and 0.8~0.9 g/cm^3^, respectively.

As to condensed bone, Ct.Ar/Tt.Ar provides a measure of the proportion of the total area that is occupied by bone, while Ct.Th describes the average thickness of condensed bone [14]. As shown in Tables 1 and 2, the femur of frog had a significantly higher Ct.Ar/Tt.Ar and Ct.Th than the rest, indicating that it was well structured with a notable amount of thick condensed bone.

As the conventional and representative indices in analyzing the microstructure of spongy bone, BV/TV is calculated upon the bone volume (BV, images from dataset with >90 grey thresholds) to the tissue volume (TV, images from dataset within the VOI), while BS/TV is the ratio of bone surface to TV [14]. Given this, BV/TV stands for the average bone content, while BS/TV shows the complexity of the bone microstructure [14]. As shown in the statistical results, rat exhibited the highest BV/TV and BS/TV in the distal part of femur, demonstrating its higher amount of bone and more complicated trabecular microstructure than that of frog and pigeon. This was further proven by its significantly higher Tb.N but lower Tb.Sp than the rest, which demonstrated that the spongy bone in the femur of rat was constructed by more complex trabeculae in a greater amount, for no statistical difference was found in Tb.Th among the studied animal bones.

### 4.4. Nanostructure

In the plasma-ashed samples, only thin platelet-like crystals were observed, in which fact was corresponding to the previous finding [8], while some “needle-like crystals” were formed because of the piling, lining and overlapping of such irregular platelets. This phenomenon was also explained as the bending, folding, curving of the thin platelet crystals [8]. This situation was worsened by the possible organic residues between crystals, which, to some extent, also led to the difficulty in the determination of the accurate crystal size. However, a rough evaluation might be made and there was no crystal larger than 100 nm. On the other hand, the reported crystal size from fish, bovine, chicken, and mouse bones was similar, about 40 nm in length and 30 nm in width, with different distribution in each animal studied [8].

By contrast, the morphology of BAp obtained by sintering was more complicated. Although only platelet-like crystals were observed in the samples from fish, rod-like crystals were also found in the rest. Assumptions may be made that, on one hand, the reaction of crystals to thermal treatment was different. It is noteworthy that such an appearance of rod-like crystals in sintered specimens seemed to correspond to the distribution of spongy bone as mentioned above. In other words, the crystals composed of condensed bone might have much slower growth than those constructing spongy bone. Given this, the former stayed as platelet-like form while the latter grew along c-axis and turned into rod during the sintering process. In addition, spongy bone was mainly found in the both ends of femurs and vertebral bodies of frog, rat, and pigeon, corresponding to the appearance of such rod-like crystals after sintering. Only platelet-like crystals were detected in the bones of fish and the middle part of femurs, for they were primarily composed of condensed bone. However, more evidence is needed to support these assumptions in further study.

Because of the rather low crystallinity as well as the possible organic residues in the specimens obtained by plasma ashing, the XRD patterns were undistinguishable except two representative peaks pertaining to calcium phosphate apatite (Figure 5(a)). By contrast, the thermal-treated samples suggested much higher crystallinity and a larger crystal size (Figure 5(b)), demonstrating the substantial undeniable effect of thermal treatment on the crystal characteristics of BAp. This said, HPO_4_^2−^ was reserved in most of the sintered specimens except frog bones (Figure 6), which was corresponding to the XRD results (Table 3), but carbonate groups that are characteristic in BAp were barely found in the sintered specimens. This was reconfirmed by the absence of the absorption peaks pertaining to carbonate groups in FTIR spectra (Figure 6(b)).

According to the peak positions of carbonate in FTIR, BAp was interpreted as a mixed-type carbonated hydroxyapatite; that is, both phosphate and hydroxyl groups were substituted by carbonate [23]. However, almost all the carbonate was removed by the thermal treatment at 800°C, leading to the undetectable peaks at the corresponding spectral sites. Moreover, hydroxyl group showed its presence only after sintering, indicating a lower content of hydroxyl in BAp than expected [26]. Additionally, different absorption bands of phosphate (*v*_4_) were found between the two fish and the other animals. This might be due to their different response to thermal treatment and lead to the unchanged irregular shape of BAp crystals after sintering. Further study is needed to clarify the exact structural and compositional cause of such different thermal reactivity of BAp from fish.

BAp is the main component in xenogeneic bone grafting materials. Its physiochemical properties, especially the rate of dissolution and solubility, play an important role in the biodegradation of bone grafting materials in a constant physiological microenvironment [27]. It is well-known that factors, such as temperature, solution composition, concentration of surface adsorbed species, size, and surface characteristics of the crystals, have a great influence on the rate of dissolution [23], while the presence of carbonate can increase the solubility of calcium phosphate apatite [28, 29]. When the biological microenvironment is assumed to be constant, (including temperature, cell activity, solution composition, and concentration of surface species), the biodegradation of grafting materials becomes faster when they have more carbonate groups contained in the lattice and also lower crystallinity and smaller crystal size. Unfortunately, all these factors have been altered to the opposite way by thermal treatments, resulting in BAp with a low content of carbonate, high crystallinity, and large crystal size and, consequently, a low solubility and a slow dissolution rate. This might probably be the main cause of the slow biodegradation of some commercial grafting materials as mentioned above. Given this, thermal treatments should be avoided in the preparation of BAp, especially for that to be used as a grafting material aiming at a relatively fast dissolution rate. The lower the temperature is, the less the negative effect on its properties would be.

## 5. Conclusion

As revealed by *μ*-CT, the animal bones studied showed different content and distribution in condensed and spongy bones, which may be composed of BAp crystals with differing thermal reactivity. Vertebrate bone-derived BAp is platelet-like rather than rod-like crystal with low crystallinity* in vivo*. The thermal treatment may cause structural and compositional changes to BAp, which should be avoided in related studies and the production of bone grafting materials.

## Figures and Tables

**Table 1:** Descriptives of Ct.Ar/Tt.Ar and Ct.Th of condensed bone.

Index	Animals	Mean	Standard deviation
Ct.Ar/Tt.Ar	Grouper	0.437	0.037
	Hairtail	0.493	0.113
	Frog	0.628	0.039
	Rat	0.445	0.035
	Pigeon	0.356	0.025

Ct.Th	Grouper	12.232	1.367
	Hairtail	16.635	1.696
	Frog	75.908	1.048
	Rat	51.658	2.145
	Pigeon	48.648	3.960

**Table 2:** Multiple comparisons for Ct.Ar/Tt.Ar and Ct.Th of condensed bone.

Index	Animals		*P* ^*^
Ct.Ar/Tt.Ar	Grouper	Hairtail	1.000
		Frog	0.001
		Rat	1.000
		Pigeon	0.437
	Hairtail	Frog	0.017
		Rat	1.000
		Pigeon	0.015
	Frog	Rat	0.001
		Pigeon	<0.001
	Rat	Pigeon	0.269

Ct.Th	Grouper	Hairtail	1.000
		Frog	<0.001
		Rat	<0.001
		Pigeon	<0.001
	Hairtail	Frog	<0.001
		Rat	<0.001
		Pigeon	<0.001
	Frog	Rat	<0.001
		Pigeon	<0.001
	Rat	Pigeon	1.000

^*^Bonferroni.

**Table 3:** Crystal characteristics of BAp obtained with thermal treatment^*^.

Sample 1 = femur/fin ray 2 = vertebral body	Identity	Cell parameters		
		*a*	*b*	*c*
Grouper 1	Ca_9_HPO_4_(PO_4_)_5_OH	9.428	9.428	6.883
	Ca_19_Mn_2_(PO_4_)_14_			

Grouper 2	Ca_9_HPO_4_(PO_4_)_5_OH	9.417	9.417	6.878
	Ca_19_Co_2_(PO_4_)_14_			

Hairtail 1	Ca_9_HPO_4_(PO_4_)_5_OH	9.433	9.433	6.880
	Ca_19_Fe_2_(PO_4_)_14_			

Hairtail 2	Ca_9_HPO_4_(PO_4_)_5_OH	9.419	9.419	6.877
	Ca_19_Fe_2_(PO_4_)_14_			

Frog 1	Ca_10_(PO_4_)_5_CO_3_(OH)F	9.415	9.415	6.879
	Mn_3_N_2_			

Frog 2	Ca_5_(PO_4_)_3_OH	9.422	9.422	6.883

Rat 1	Ca_9_HPO_4_(PO_4_)_5_OH	9.419	9.419	6.878
	Ca_19_Co_2_(PO_4_)_14_			

Rat 2	Ca_9_HPO_4_(PO_4_)_5_OH	9.435	9.435	6.884
	Ca_19_Ni_2_(PO_4_)_14_			

Pigeon 1	Ca_9_HPO_4_(PO_4_)_5_OH	9.417	9.417	6.880
	Ca_19_Co_2_(PO_4_)_14_			

Pigeon 2	Ca_9_HPO_4_(PO_4_)_5_OH	9.422	9.422	6.881
	Ca_19_Co_2_(PO_4_)_14_			

HAp	Ca_5_(PO_4_)_3_OH	9.418	9.418	6.884

HAp-HPO_4_	Ca_9_HPO_4_(PO_4_)_5_OH	9.441	9.441	6.881

^*^Obtained by software *Jade*.

## References

[1] Cordaro L., Bosshardt D. D., Palattella P., Rao W., Serino G., Chiapasco M. (2008). Maxillary sinus grafting with Bio-Oss or Straumann Bone Ceramic: histomorphometric results from a randomized controlled multicenter clinical trial.

[2] Cannizzaro G., Felice P., Leone M., Viola P., Esposito M. (2009). Early loading of implants in the atrophic posterior maxilla: lateral sinus lift with autogenous bone and Bio-Oss versus crestal mini sinus lift and 8-mm hydroxyapatite-coated implants. A randomised controlled clinical trial.

[3] Piattelli M., Favero G. A., Scarano A., Orsini G., Piattelli A. (1999). Bone reactions to anorganic bovine bone (Bio-Oss) used in sinus augmentation procedures: a histologic long-term report of 20 cases in humans.

[4] Liu Q., Huang S., Matinlinna J. P., Chen Z., Pan H. (2013). Insight into biological apatite: physiochemical properties and preparation approaches.

[5] Pearce A. I., Richards R. G., Milz S., Schneider E., Pearce S. G. (2007). Animal models for implant biomaterial research in bone: a review.

[6] Duda M., Pajak J. (2004). The issue of bioresorption of the Bio-Oss xenogeneic bone substitute in bone defects.

[7] Eppell S. J., Tong W., Lawrence Katz J., Kuhn L., Glimcher M. J. (2001). Shape and size of isolated bone mineralites measured using atomic force microscopy.

[8] Kim H.-M., Rey C., Glimcher M. J. (1995). Isolation of calcium-phosphate crystals of bone by non-aqueous methods at low temperature.

[9] Jackson S. A., Cartwright A. G., Lewis D. (1978). The morphology of bone mineral crystals.

[10] Figueiredo M., Fernando A., Martins G., Freitas J., Judas F., Figueiredo H. (2010). Effect of the calcination temperature on the composition and microstructure of hydroxyapatite derived from human and animal bone.

[11] Wang X.-Y., Zuo Y., Huang D., Hou X.-D., Li Y.-B. (2010). Comparative study on inorganic composition and crystallographic properties of cortical and cancellous bone.

[12] Ito M. (2011). Recent progress in bone imaging for osteoporosis research.

[13] (2010).

[14] Bouxsein M. L., Boyd S. K., Christiansen B. A., Guldberg R. E., Jepsen K. J., Müller R. (2010). Guidelines for assessment of bone microstructure in rodents using micro-computed tomography.

[15] Wagner D. O., Aspenberg P. (2011). Where did bone come from? An overview of its evolution.

[16] Janus A. M., Faryna M., Haberko K., Rakowska A., Panz T. (2008). Chemical and microstructural characterization of natural hydroxyapatite derived from pig bones.

[17] Ooi C. Y., Hamdi M., Ramesh S. (2007). Properties of hydroxyapatite produced by annealing of bovine bone.

[18] Murugan R., Ramakrishna S., Rao K. P. (2006). Nanoporous hydroxy-carbonate apatite scaffold made of natural bone.

[19] Pan H.-B., Li Z.-Y., Wang T. (2009). Nucleation of strontium-substituted apatite.

[20] Danilchenko S. N., Koropov A. V., Protsenko I. Y., Sulkio-Cleff B., Sukhodub L. F. (2006). Thermal behavior of biogenic apatite crystals in bone: an X-ray diffraction study.

[21] Rhee S.-H., Park H. N., Seol Y.-J., Chung C.-P., Han S. H. (2006). Effect of heat-treatment temperature on the osteoconductivity of the apatite derived from bovine bone.

[22] Pang Y. X., Bao X. (2003). Influence of temperature, ripening time and calcination on the morphology and crystallinity of hydroxyapatite nanoparticles.

[23] Elliott J. C. (1994).

[24] Kuhn L. T., Grynpas M. D., Rey C. C., Wu Y., Ackerman J. L., Glimcher M. J. (2008). A comparison of the physical and chemical differences between cancellous and cortical bovine bone mineral at two ages.

[25] Tong W., Glimcher M. J., Katz J. L., Kuhn L., Eppell S. J. (2003). Size and shape of mineralites in young bovine bone measured by atomic force microscopy.

[26] Rubin M. A., Jasiuk I. (2005). The TEM characterization of the lamellar structure of osteoporotic human trabecular bone.

[27] Vallet-Regí M. (2008).

[28] Chen Z.-F., Darvell B. W., Leung V. W.-H. (2004). Hydroxyapatite solubility in simple inorganic solutions.

[29] Pan H. B., Darvell B. W. (2010). Effect of carbonate on hydroxyapatite solubility.

